# Production of Biologically Active Cecropin A Peptide in Rice Seed Oil Bodies

**DOI:** 10.1371/journal.pone.0146919

**Published:** 2016-01-13

**Authors:** Laura Montesinos, Mireia Bundó, Esther Izquierdo, Sonia Campo, Esther Badosa, Michel Rossignol, Emilio Montesinos, Blanca San Segundo, María Coca

**Affiliations:** 1 Institute of Food and Agricultural Technology-CIDSAV-XaRTA, University of Girona, Girona, Spain; 2 Centre for Research in Agricultural Genomics (CRAG), CSIC-IRTA-UAB-UB, Edifici CRAG, Campus de la UAB, Bellaterra, Barcelona, Spain; 3 Mass Spectrometry Proteomics Platform, Biochimie et Physiologie Moléculaire des Plantes, SupAgro/INRA/CNRS/UMII/UMR 5004, Montpellier, France; Institute of Genetics and Developmental Biology, Chinese Academy of Sciences, CHINA

## Abstract

Cecropin A is a natural antimicrobial peptide that exhibits fast and potent activity against a broad spectrum of pathogens and neoplastic cells, and that has important biotechnological applications. However, cecropin A exploitation, as for other antimicrobial peptides, is limited by their production and purification costs. Here, we report the efficient production of this bioactive peptide in rice bran using the rice oleosin 18 as a carrier protein. High cecropin A levels were reached in rice seeds driving the expression of the chimeric gene by the strong embryo-specific *oleosin 18* own promoter, and targeting the peptide to the oil body organelle as an oleosin 18-cecropin A fusion protein. The accumulation of cecropin A in oil bodies had no deleterious effects on seed viability and seedling growth, as well as on seed yield. We also show that biologically active cecropin A can be easily purified from the transgenic rice seeds by homogenization and simple flotation centrifugation methods. Our results demonstrate that the oleosin fusion technology is suitable for the production of cecropin A in rice seeds, which can potentially be extended to other antimicrobial peptides to assist their exploitation.

## Introduction

Antimicrobial peptides (AMPs) are short, predominantly cationic, and amphipathic compounds that exhibit rapid, potent and long-lasting activity against a wide range of microbes, including bacteria, fungi, viruses and protozoa, and even neoplastic cells [1,2]. In addition to natural AMPs, many synthetic AMPs have been designed with potentially superior properties, including stability and specificity [3–5]. Some of these synthetic peptides are based on cecropin A (CecA), a linear and cationic AMP isolated from insect haemolymph, with potent lytic activity against important bacterial and fungal phytopathogens, and great biotechnological potential [3,6–8] These natural and synthetic antibiotics are envisaged as new agents for crop protection, for food conservation, and for cosmetics and clinical therapies [4,9–15]. However, their application has been limited due to the high cost of chemical synthesis and the low yield obtained via purification from natural sources.

The use of plants as biofactories for AMPs might represent an economical and safe alternative. Although, the production of these bioactive peptides in plant systems has been challenging due to either instability or degradation in plant tissues [14,16,17], or to phytotoxicity that results in a penalty on plant performance [18–21]. Rice seeds offer unique opportunities as bioreactors since the rice gene transfer technology is well developed, cropping conditions are easy and well-established worldwide, and high grain yield can be obtained [22,23]. The production of several recombinant proteins and peptides has been successfully accomplished in transgenic rice seeds, including vaccines [24–27], hormones [28], antibodies [29], and other pharmaceutical peptides [30–34]. Interestingly, our group has demonstrated that transgenic rice plants expressing a codon-optimized synthetic *CecA* gene driven by endosperm-specific promoters accumulate CecA peptide in seed storage protein bodies without a negative effect on plant performance [35]. This evidence suggested that limiting the accumulation to storage organs such as rice seeds is a suitable production strategy for AMPs.

All the recombinant proteins/peptides produced in rice seeds have been accumulated into protein bodies (PBs), but there is still the possibility of targeting accumulation onto oil bodies (OBs). These are small spherical discrete intracellular organelles (0.5–2 μm) that serve as lipid reservoirs for seed germination and seedling growth prior to photosynthetic establishment [36–38]. They consist of a neutral lipid core surrounded by a monolayer of phospholipids coated with specific proteins, predominantly oleosins, and some other minor proteins such as caleosins and steroleosins [38,39]. Oleosins are lipophilic small proteins with a unique secondary structure consisting in a central hydrophobic domain highly conserved that penetrates through the phospholipid monolayer anchoring them to the OB; and with two variable amphipatic N and C terminal domains covering the OB surface [40,41]. The physicochemical properties of oleosins and their association with OBs have led to their use as carriers of recombinant proteins. This use was first demonstrated with the production of a fusion protein between the oleosin and the β-glucuronidase enzyme in the transgenic *Brassica napus* seed OBs [42]. Later, this technology was developed to produce pharmaceutical proteins, including the 6.9 kDa hirudin in *Brassica* [43], the 28 kDa apolipoprotein AI in safflower [44], the 22 kDa growth hormone [45] and a 5.7 kDa insulin in *Arabidopsis* [46]. Nevertheless, little attention has been paid to the oleosin fusion technology for the production of AMPs, and it is yet unknown the effect of the fusion of such strongly cationic and amphipathic bioactive peptides in the behavior of OBs, and in the seed physiology. The present study was undertaken to explore the feasibility of using rice seed OBs for the accumulation and subsequent purification of AMPs *in planta*. CecA was chosen as the model AMP to be produced using the OB targeting approach. Here, we report that CecA can be produced and accumulated in rice OBs as an Oleosin18-CecA fusion protein, and that bioactive CecA can be easily purified from the recombinant OBs. Interestingly, CecA accumulation in seed OBs had no deleterious effects on the normal growth and development of the rice plant, as well as on grain yield.

## Materials and Methods

### Preparation of plant expression vectors

**Fig 1:**
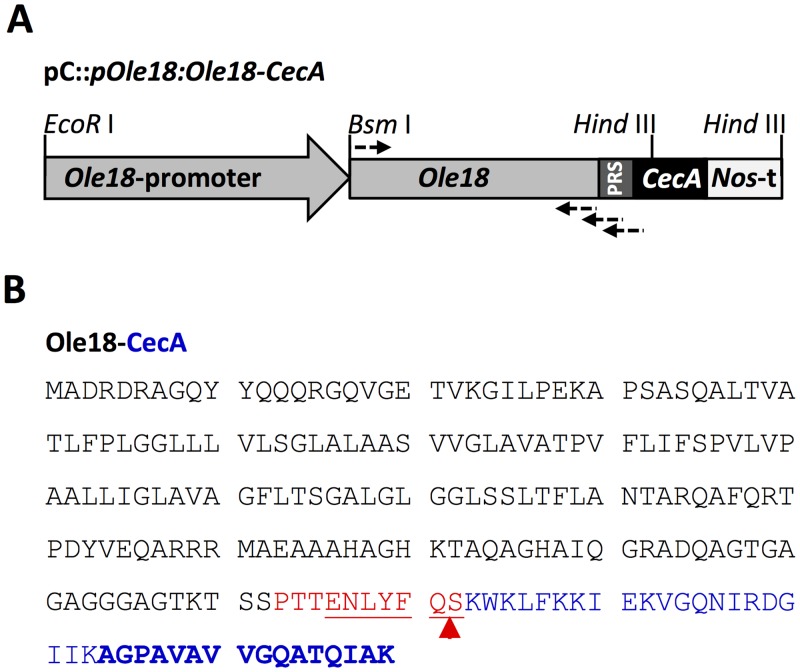
Chimeric gene used for the production of cecropin A as an oleosin fusion protein in rice seeds. (A) Schematic diagram of the construct used for rice transformation. Relevant restriction enzyme sites and primers (arrows) used for cloning are indicated. (B) Amino acid sequence of the fusion protein Ole18-CecA containing the rice 18 kDa oleosin (black) and the cecropin A peptide (blue) linked through a TEV protease recognition site (PRS, red). Red arrow indicates TEV protease cleavage site.

A plant expression vector containing a chimeric gene encoding the Ole18-CecA fusion protein under the control of the *Ole18* promoter was prepared for rice transformation (Fig 1A). The *Ole18* promoter (1139 pb) including the 5´-untranslated region (61 pb) was amplified by PCR from genomic rice DNA (*Oryza sativa* ssp. *indica* cv. IR36) using primers (S1 Table) designed according to the nucleotide sequence at the GenBank database (AY427563) for the Nipponbare *japonica* rice cultivar.

The fusion gene was obtained by three consecutive rounds of PCR amplification. In the first one, the *Ole18* full-length cDNA was amplified from AK243015 clone (Rice Genome Research Center) using the primers Ole18cds1 and Ole18cds2 (S1 Table), which introduced a *Bsm*I site at the 5´ end and eliminated the stop codon at the 3´ end, respectively. In the second round, an *Ole18* fragment extended with the nucleotides encoding the Tobacco Etch Virus (TEV) protease recognition site was amplified using the Ole18cds1 and Ole18cds3 primers. Finally, a third PCR reaction with the Ole18cds1 and the Ole18cds4 primers extended the fragment with the first 12 nucleotides of *CecA* containing a *Hind*III site. After cloning into the pGEM-t Easy vector (Promega), the *Bsm*I-*Pst*I fragment was inserted downstream of the *Ole18* promoter in the pGEM-t Easy vector. Next, the *Ole18* promoter and the partial fusion gene *Ole18-CecA* fragment was cloned into the plant expression vector pCAMBIA1300 as an *EcoR*I-*Hind*III fragment. Finally, the rest of *CecA* gene and the *Nopaline synthase* (*Nos*) terminator signal were introduced into this vector as a *Hind*III fragment to obtain the final vector pC::*pOle18*:*Ole18-CecA*. The DNA fragment containing the *CecA* and the *Nos* terminator was obtained from a previously described plasmid [19]. The construct was then verified by nucleotide sequencing.

### Production of transgenic rice plants

Transgenic rice lines (*O*. *sativa* cv. Ariete and cv. Senia) were produced by *Agrobacterium*-mediated transformation of embryonic callus derived from mature embryos, as described previously [47]. Transgene insertion was confirmed in the regenerated plants by PCR analysis using leaf genomic DNA as the template. The positive plants were grown under containment greenhouse conditions to obtain homozygous transgenic lines in the T2 generation. Homozygous lines were identified by segregation of hygromycin resistance afforded by the *htpII* marker gene in the T-DNA region of pCAMBIA1300. The transgene copy number was estimated by quantitative PCR analysis of the T2 homozygous lines as previously described [20,35,48]. Rice plants transformed with the empty vector (pCAMBIA 1300) were also produced as a control for this study. All rice plants were grown at 28°C±2°C under a 14 h/10 h light/dark photoperiod.

### In situ immunodetection of CecA in whole seeds

CecA accumulation in the transgenic rice seeds was analysed by *in situ* immunodetection using specific anti-CecA antibodies [19] and the fluorescent labelled AlexaFluor488 anti-rabbit IgG as secondary antibodies (Molecular Probes, 1:5000 dilution) as previously described [35].

### OBs purification and immunodetection of OB proteins

OBs were easily isolated by flotation centrifugation on a two layer sucrose density gradient as previously described with some modifications [40]. Basically, the protocol was adapted to small amounts of starting material. Normally, 10 dehulled mature seeds (around 0.2 g) were imbibed for two hours in water and homogenized in grinding buffer (2 ml of 0.6 M sucrose in phosphate buffer pH 7.5) using a mortar and pestle. The homogenate was then layered under equal volume of flotation buffer (0.4 M sucrose in phosphate buffer pH 7.5) and centrifuged at 10000 *x g* for 20 min. The floating fat pad was collected and resuspended in 1ml grinding buffer containing 2 M NaCl, and then overlaid with an equal volume of floating buffer. After centrifugation, the OBs were recovered at the top of the supernatant phase and resuspended in 100 μl of 0.2 M sucrose in phosphate buffer pH 7.5. The integrity of isolated OBs was tested by selective staining with Nile red (1 ng/ml, Sigma) and fluorescence microscopy visualization. Immunofluorescence microscopy of OBs was performed using the rabbit anti-CecA antibodies (1:200 dilution), and the fluorescent labelled AlexaFluor488 anti-rabbit IgG as secondary antibodies (Molecular Probes, 1:5000 dilution), and visualized with a confocal laser scanning microscope (Leica TCS-SP5II). *In vivo* OBs were also visualized by confocal microscopy in whole mount longitudinal embryo sections infiltrated with Nile red.

Immunoblot analysis of OB-associated proteins was done after their solubilization in loading buffer (from 5 μl of isolated OBs), separationby SDS-PAGE,transferto nitrocellulose membranes and immunoreactions with antibodies against the CecA (1:500 dilution), the rice Ole18 (1:2000 dilution) and the sesame caleosin (1:1000 dilution) [49]. Rabbit polyclonal antibodies against the rice Ole18 were produced at the Laboratory Animal Facilities (registration number: B9900083) of the Center for Research and Development (CID) from the Spanish National Research Council (CSIC), in strict accordance with the bioethical principles established by the Spanish legislation following international guidelines. The protocol was approved by the Committee on Bioethics of Animal Experimentation from CID and by the Department of Agriculture, Livestock, Fisheries, Food and Environment of the Government of Catalonia (permit number DAAM:7461). All efforts were made to minimize suffering of the animals. For the antibodies production, the Ole18 protein was purified from rice OB proteins resolved by SDS-PAGE using the method described by [28]. Three weekly injections of the recovered Ole18 protein (50 μg each) were applied to rabbits, and one week after they were bled to obtain the Ole18 antiserum.

The amount of the accumulated CecA per seed was estimated by quantification of signal intensities on Western-blot analysis in comparison with known amount of synthetic CecA peptide. Signal intensities were quantified using the MultiGauge v3.0 (Fujifilm) software. Quantification was performed in 3 independent experimental replicas for 11 independent transgenic lines.

### Purification of CecA from OBs

CecA was recovered from the OBs containing the Ole18-CecA polypeptide by digestion with the TEV protease (Sigma, 1:100 dilution). Proteolytic digestion was conducted overnight at room temperature in OB resuspension buffer (100 μl of 0.25 M sucrose in 10 mM phosphate buffer pH7.5) supplemented with 50 mM NaCl, 1mM DTT and 0.5 mM EDTA. TEV efficiency was estimated based on the disappearance of the Ole18-CecA signal on immunoblot analysis by quantication of the signal intensities using the MultiGauge v3.0 (Fujifilm) software. Then, digested OBs were centrifuged at 10000 *x g* for 20 min, and CecA was recovered in the soluble fraction after separation of OBs on the top. Protein concentration was determined by UV absorbance at 280 nm in comparison with the wild-type fraction supplemented with known amount of synthetic CecA. For Western-blot analysis, proteins in the soluble fraction (90 μl) were concentrated by acetone precipitation, and separated togheter with those in floating fractions (10 μl) by a Tricine-SDS-PAGE.

### Mass Spectrometry Analysis

OB samples and soluble protein fractions obtained by centrifugation after TEV digestion of OBs were diluted in 6 volumes of 25 mM NH_4_HCO_3_ with 10 μl of trypsin (0.1 μg/μl) and incubated overnight at 37°C. Increasing amounts of synthetic CecA were treated similarly (1, 10 and 50 ng). Digested samples were desalted on Sep-Pak cartridges (Waters) and eluted with 70% acetonitrile containing 0.1% formic acid. Eluted samples were dried and dissolved in 0.1% formic acid before LC-MS analysis. The protein digests were analysed using a QTOF mass spectrometer (Maxis Impact, Bruker Daltonik GmbH) interfaced with a nano-HPLC Ultimate 3000 (Dionex). LC-MS/MS analysis was performed in the full scan MS/MS mode as previously described [35]. For fast monitoring, the CecA containing samples were analyzed in MRM mode (Multiple Reaction Monitoring) on a 4000 QTRAP LC-MS/MS hybrid triple quadruple/linear ion trap mass spectrometer using a microSpray source (AB/MDS Sciex) coupled to an Agilent 1200 nanoflow HPLC. Peptide samples were separated on a nano-flow reverse-phase column (Agilent Zorbax 300 SB-C18) and eluted with a linear gradient from 10 to 50% acetonitrile in 0.1% formic acid. The instrument was operated in positive ion mode and the source parameters were: 2500 V ion spray voltage, 20 psi curtain gas, 150°C interface heater temperature, 140 V declustering potential, collision-activated dissociation pressure set on high, and Q1 and Q3 set to unit resolution (0.6–0.8 Da full width at half-height). Based on full scan mode results, the three most intense transitions were chosen to follow the CecA tryptic peptide AGPAVAVVGQATQIAK. A dwell time of 100ms and a collision energy of 36 were used for all the transitions.

### Antimicrobial assays

The antimicrobial activity of the *in planta* produced CecA was assessed against *D*. *dadantii* (isolate 1552 10.1), and compared to that of the synthetic CecA. For antimicrobial assays, bacterial cultures (10^5^ cells in 50 μl of sterile water) were incubated for 2 h at 28°C with the synthetic CecA or purified fractions (50, 40, 20 or 10 μl) from wild-type or transgenic rice seeds (100 μl total volum). All samples were prepared in triplicate.Then, aliquots of ten-fold serial dilutions were plated on LB-agar media and grown for 2 days at 28°C to determine the viable cells in the spot assay and in the activity titration assay Bioassays were repeated at least twice.

## Results

### Generation and characterization of transgenic rice plants

Two distinct oleosin isoforms with molecular masses of 18 and 16 kDa are present and accumulated at comparable levels in rice OBs [40]. Both of them can potentially be used for the production of an oleosin-CecA fusion protein. We selected the 18 kDa oleosin (Ole18) isoform as the carrier protein for CecA production in rice seeds. A plant expression vector containing the *Ole18-CecA* recombinant gene was prepared (Fig 1A). The expression of the recombinant gene was under the control of the *Ole18* promoter, which was obtained from the indica IR36 cultivar after several attempts to isolate the promoter from japonica cultivars without success. The DNA sequence of the isolated *Ole18* promoter has several nucleotide changes compared to the japonica Nipponbare cultivar in the database (S1 Fig), which might be explained by differences among the two rice varieties. The sequence encoding the recognition site for the Tobacco Etch Virus (TEV) protease was used to fuse the Ole18 polypeptide to the CecA peptide (Fig 1B).

Transformation of embryogenic rice calli was performed via *Agrobacterium tumefaciens*. The hygromycin resistance gene was used as the selectable marker. Sixteen and twenty-six transgenic hygromycin resistant plants (cv. Ariete and Senia, respectively) were regenerated carrying the *pOle18*:*Ole18-CecA* transgene from two independent transformation assays in the two different japonica backgrounds. Transgene integration was verified by PCR analysis (S2 Fig). No apparent adverse effects on the plant phenotype during the vegetative growth under greenhouse conditions were observed for the transgenic regenerated plants. Five and six independent lines from Ariete and Senia cultivars, respectively, and carrying one single copy of the *pOle18*:*Ole18-CecA* transgene, as determined by quantitative PCR, were selected to obtain the T2 homozygous progeny plants. The stability of the transgene integration and inheritance was monitored across generations by the hygromycin resistance phenotype encoded in the T-DNA.

### Accumulation of cecropin A in rice OBs

**Fig 2:**
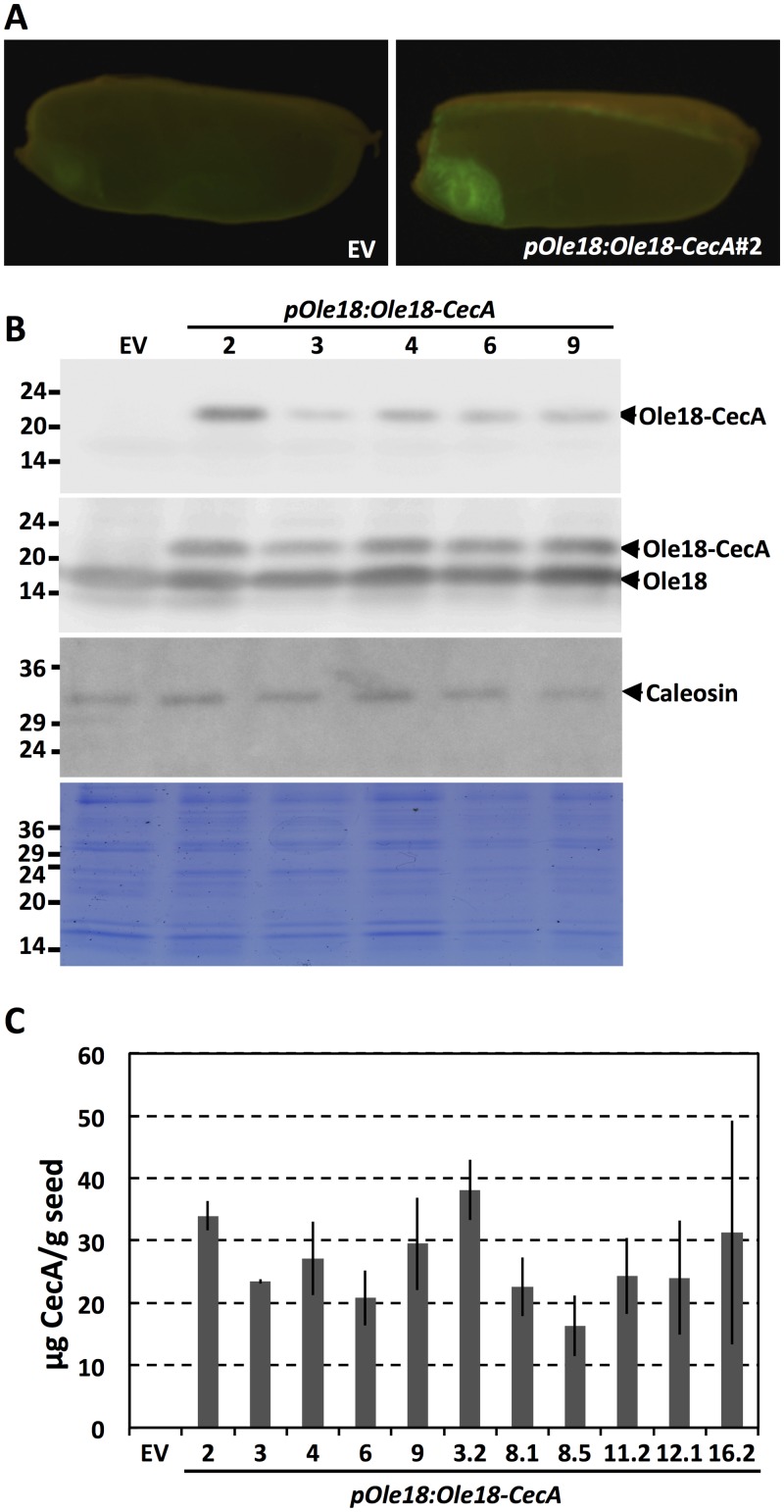
Accumulation of the Ole18-CecA fusion protein in rice seeds. (A) *In situ* immunolocalization of CecA in *pOle18*:*Ole18-CecA* transgenic seeds. Immunoreaction was detected using a fluorescent-labeled secondary antibody. (B) Western blot analysis of OB proteins purified from seeds of the indicated Ariete transgenic lines using anti-cecropin A, anti-oleosin18 and anti-caleosin antibodies (from top to bottom, respectively). Lower panel correspond to the Coomassie Blue stained SDS gel. Molecular weight markers are indicated on the left in kDa. (C) Cecropin A accumulation in seeds from the Ariete (2, 3, 4, 6, 9) and Senia (3.2, 8.1, 8.5, 11.2, 12.1, 16.2) transgenic lines (T3 homozygous) as estimated by immunoblot analysis of OB protein extracts in comparison with known amounts of synthetic cecropin A. Values correspond to the mean of at least 3 independent assays, and bars to the SD.

Accumulation of the transgene product was analyzed by *in situ* immunodetection of CecA in the rice seeds. As shown in Fig 2A, CecA was detected in the seed embryo and aleurone layers of transgenic rice plants containing the *pOle18*:*Ole18-CecA* transgene, but not in the empty vector or wild-type seeds. This result indicates that the *Ole18* promoter drives gene expression to embryos and aleurone layers, and excludes it from endosperm tissues of rice seeds.

OBs and associated proteins can be easily fractionated from other seed components by flotation centrifugation. To confirm that CecA was produced as an oleosin fusion protein and retains the natural oleosin targeting to OBs, we purified OBs from mature transgenic seeds. OBs were obtained by two consecutive cycles of two layer flotation, and their associated proteins solubilized, separated by SDS gel electrophoresis and probed with either anti-CecA or anti-Ole18 antibodies. As shown in Fig 2B, a polypeptide corresponding to the 23.2 kDa molecular mass predicted for the fusion protein (18 kDa oleosin + 1.2 kDa TEV protease recognition site + 4 kDa CecA) was clearly detected by the anti-CecA antibodies in the *pOle18*:*Ole18-CecA* OB proteins, which was absent in empty vector OB proteins. This recombinant polypeptide was also immunoreacting with the anti-Ole18 antibodies and detected as an additional band to the 18 kDa oleosin band present in all the rice OBs analyzed. The fusion protein was detected in all the produced transgenic *pOle18*:*Ole18-CecA* Ariete (Fig 2B) and Senia (S3 Fig) lines. The accumulation of this fusion protein appears not to alter the protein profile of OBs as visualized by the Coomasie-blue staining; or the accumulation of the caleosin, another integral OB protein, which accumulated at similar levels in *pOle18*:*Ole18-CecA* and empty vector OBs (Fig 2B, bottom panels). These results demonstrate that the Ole18-CecA fusion protein is produced and accumulated in the OBs of the transgenic rice seeds.

The amount of the produced CecA in rice seeds was estimated in the T3 homozygous lines. For this, the accumulation of CecA fusion protein was determined by Western blot analysis and quantification of band intensities in comparison with known amounts of CecA synthetic peptide. As shown in Fig 2C, similar values were obtained for the different transgenic lines in the two different backgrounds, ranging from 16 to 38 μg of CecA per gram of seed weight.

### Accumulation of Ole18-CecA affects the size and the surface properties of OBs

**Fig 3:**
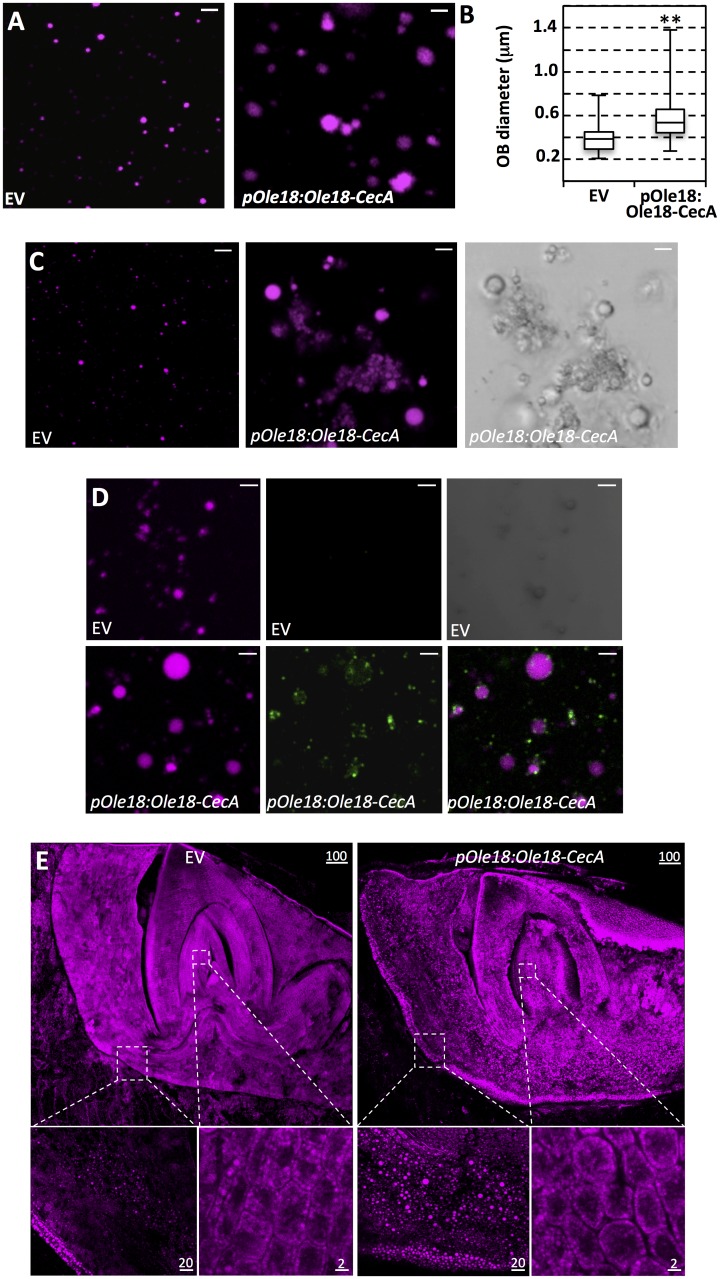
The Ole18-CecA fusion protein localizes in rice OBs modifying their size and surface properties. (A) Morphology of OBs purified from seeds carrying the empty vector (EV) or the *pOle18*:*Ole18-CecA* transgene. Images correspond to single scanned confocal microscopy slides of Nile red stained OBs. (B) Increased size of *pOle*:*Ole18-CecA* OBs. Box plot of the median sizes of 3 different OB preparations per line (n = 25 per preparation). Boxes represent the 2^nd^ and 3^rd^ quartiles of the data, whereas whiskers indicate the minimum and maximum measured OB diameters. Asterisks denote statistically significant differences (**p<0.01, ANOVA) (C) Aggregation of OBs isolated from *pOle*:*Ole18-CecA* transgenic seeds. Confocal and light (left panel) microscopy images of Nile red stained OBs after incubation for 1 hour at room temperature. (D) Immunolocalization of CecA in *pOle18*:*Ole18-CecA* OBs. CecA was immunodetected and visualized in green using an AlexaFluor488-conjugated secondary antibody. (E) Confocal microscopy images of mature embryos stained with Nile red. Scale bars correspond to 2 μm (A-D), or 2, 20 or 100 μm as indicated (E).

Next, we examined whether the accumulation of the Ole18-CecA fusion protein has an effect on the OB structure. For this, OBs were isolated from transgenic and wild-type seeds, stained with Nile red (neutral lipid stain), and visualized under confocal microscopy. OBs obtained from the seeds accumulating the recombinant protein were spherical with normal appearance. However, they showed a larger size than the ones obtained from empty vector seeds (Fig 3A). OB diameters were measured on transmission images to avoid staining interferences, and a statistically significant increase in size was quantified for OBs accumulating the Ole18-CecA (Fig 3B). Several reports described that the content of oleosin determines the size of the OB, most of them showing that reduction of the oleosin content results in larger OBs [50–52]. Our observations suggest that the accumulation of the recombinant Ole18 fusion protein increases the size of the rice seed OBs.

During the course of these analyses, we noticed that isolated OBs containing the Ole18-CecA protein tend to aggregate with time but without coalescing (Fig 3C). Under the same experimental conditions, this behavior was not observed for the empty vector OBs. The aggregation tendency of the OBs containing the Ole18-CecA might be caused by the presence of the CecA at the surface of the OBs. CecA is a highly positive charged peptide at physiological pH, which can attenuate the electronegative repulsion among the organelles. Indeed immunofluorescence microscopy experiments confirmed the presence of CecA at the OB surface. As shown in Fig 3D, specific CecA immunodecoration was detected as green fluorescence surrounding the Nile red stained core of OBs obtained from the *pOle18*:*Ole18-CecA* (lower panels), which was not observed on empty vector OBs (upper panels). Therefore, the accumulation of the Ole18-CecA protein increases the size and confers different surface properties to isolated OBs. These OB changes seems not to have a drastic impact in *in vivo* OBs or embryonic cells, as observed by confocal microscopy analysis of Nile red stained mature embryos (Fig 3E).

### Purification and identification of *in planta* produced cecropin A

**Fig 4:**
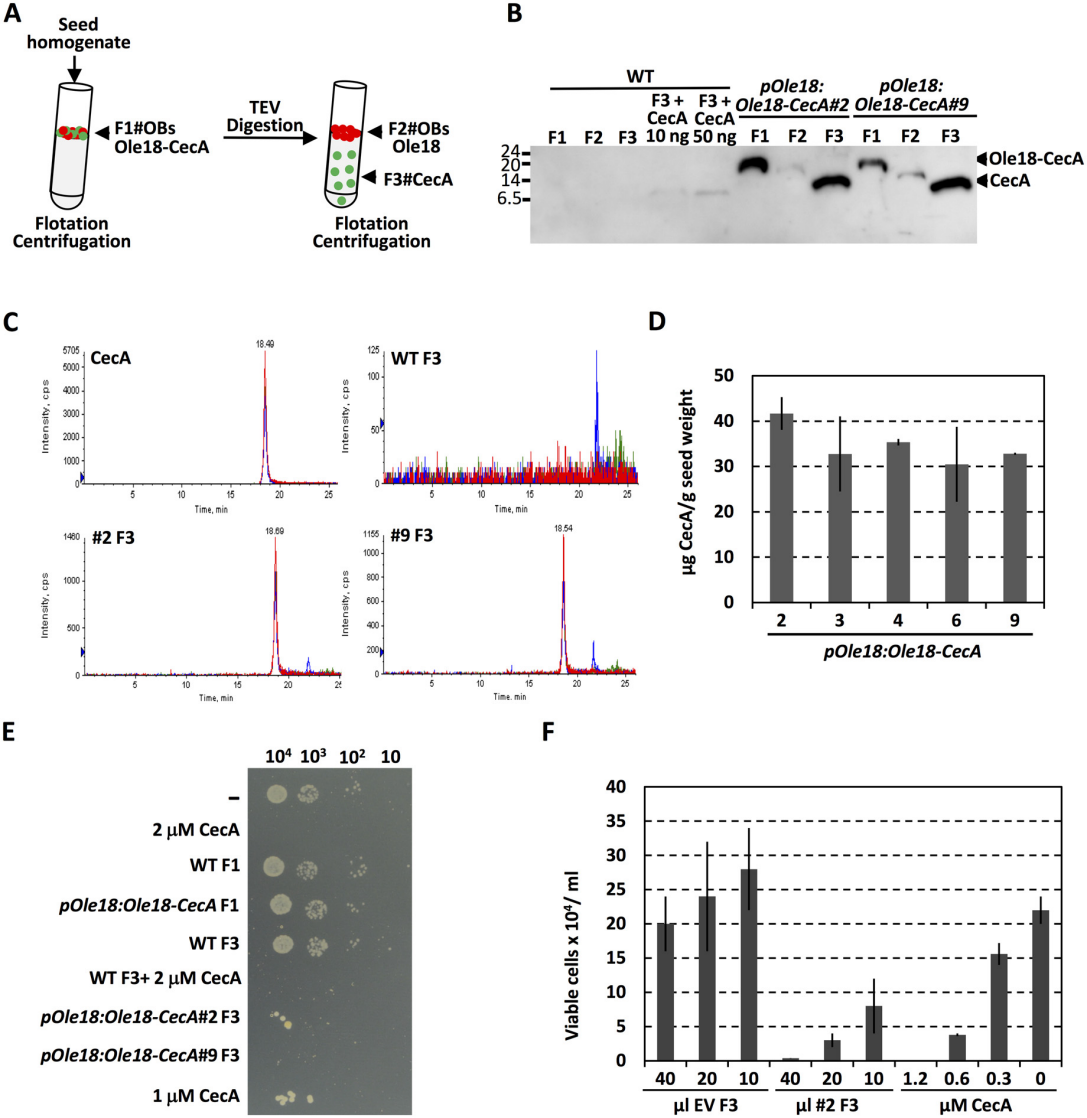
Purification of active cecropin A from *pOle18*:*Ole18-CecA* plant seeds. (A) Schematic purification procedure of CecA. (B) Immunoblot analysis of purified fractions from wild-type (WT) and two independent *pOle18*:*Ole18-CecA* transgenic lines (10 seeds per line). As a control, synthetic cecropin A (10 and 50 ng of CecA) was added to one WT F3 fraction. The proteins in the F3 fractions were concentrated by acetone precipitation, and together with the F1 and F2 fraction proteins, were resolved in Tricine-SDS-PAGE and immunodetected with anti-CecA antibodies. (C) MRM chromatograms of CecA synthetic peptide, WT and *pOle18*:*Ole18-CecA* (lines #2 and #9) F3 fractions. The MS/MS transitions monitored were 740.9/816.4, 740.9/915.5 and 740.9/1085.6. (D) Amount of CecA in F3 fractions as determined by UV absorbance (280 nm) in comparison with known amounts of synthetic CecA added to wild-type F3 fractions. (E) Antimicrobial activity of purified fractions against *Dickeya dadantii* bacterial cells. Aliquots (3 μl) of ten-fold serial dilutions of bacterial cells (5 x 10^4^) incubated for 2h with synthetic CecA or with purified fractions (5 μl of F1 or F2, or 50 μl of F3 fractions) from WT or from the indicated *pOle18*:*Ole18-CecA* transgenic lines were plated on LB-agar media and grown for 2 days. (F) *D*. *dadantii* viable cells after 2h incubation with the indicated amounts of F3 fractions from empty vector (EV) or *pOle18*:*Ole-CecA* (line #2) compared to synthetic CecA at indicated concentrations. Mean values of two replicates and SD are shown.

Once shown that the Ole18-CecA fusion protein accumulates in rice seed OBs, we assessed the efficiency of CecA extraction and recovery from plant material. A schematic diagram of the purification procedure used is shown in Fig 4A. OBs and associated proteins were separated from the rest of the seed components using flotation centrifugation (F1 fraction). Since the fusion protein contains a TEV recognition site between the Ole18 and CecA polypeptides, intact OBs were subjected to TEV proteolytic digestion to release CecA peptide (Fig 1B). The released CecA was then recovered at high purity in the soluble fraction (F3 fraction) by separating from OBs on the supernatant upon one more centrifugation (F2 fraction). The immunoblot analysis of the fractions from two independent *pOle18*:*Ole18-CecA* lines and wild-type seeds is shown in Fig 4B. The fusion protein clearly detected in F1 fractions from the transgenic lines nearly disappeared in F2 fractions, showing almost complete release of CecA from the fusion protein by TEV digestion, and indicating a high efficiency of the TEV proteolysis on intact OBs. The TEV efficiency varied between 70 to 100% in different assays and different lines, in most of the cases near 90%, as estimated by quantification of the disappearance of the Ole18-CecA band signals on Western-blot analysis. A polypeptide with a similar mobility to the synthetic CecA added to wild-type F3 fraction (F3+CecA in Fig 4B) was detected in the transgenic F3 fractions, demonstrating that CecA can be recovered after cleavage.

The presence of CecA in the soluble fractions (F3) was confirmed by MS/MS analysis, which identified the tryptic peptide AGPAVAVVGQATQIAK (monoisotopic mass 740.92) corresponding to the C-terminal of CecA (Fig 1B). Additionally, F3 fractions were fast monitored by MRM analysis (Fig 4C). An elution peak was detected in the two *pOle18*:*Ole18-CecA* samples similar to the one detected for the synthetic CecA standard, and absent in the wild-type sample. These results demonstrated that CecA was produced and recovered following simple purification schemes from the transgenic rice seeds. The amount of recovered CecA was in the range of 30–40 μg per gram of rice seed as determined by UV absorbance in comparison with synthetic CecA (Fig 4D).

### Plant-produced cecropin A exhibits biological activity

The antimicrobial activity of the *in planta* produced CecA was evaluated against the phytopathogenic bacteria *Dickeya dadantii*. OB fractions (F1) and soluble fractions (F3) from wild-type and transgenic lines were assayed for bactericidal activity using a contact killing test, and compared to the synthetic CecA. As shown in Fig 4E, the wild-type and transgenic OBs (F1 fractions) were not active suggesting that the fusion protein is inactive. However, the F3 fractions from *pOle18*:*Ole18-CecA* lines showed a potent lytic activity against bacterial cells, as observed for the synthetic CecA. No activity was detected in the wild-type F3 fraction, which became active upon addition of synthetic cecropin A (WT F3 + CecA in Fig 4E). These results showed that the plant CecA was as cytotoxic for *D*. *dadantii* as the synthetic CecA. They also demonstrated that the *in planta* produced CecA was functional but requires to be released from the carrier Ole18 protein for activity. Next, the purified fractions (F3) were titrated for antimicrobial activity against *D*. *dadantii* in comparison with a calibration curve of synthetic CecA. As shown in Fig 4F, the synthetic CecA at low concentrations reduced the viability of bacterial cells, causing complete lost of viability at 1.2 μM concentration. Interestingly, similar bactericidal activity was detected when adding 40 μl of F3 fractions from the *pOle18*:*Ole18-CecA* line 2, whereas no antibacterial activity was detected for the same amounts of EV F3 fractions under the assay conditions. Taking into account that 20 μl of F3 fractions obtained from line 2 seeds showed similar activity than 0.6 μM of synthetic CecA, a similar concentration of active cecropin A was estimated meaning that more than 6 μg of active CecA can be recovered from gram of rice seeds.

### Phenotypic effects of *Ole18-CecA* expression in rice plants

**Fig 5:**
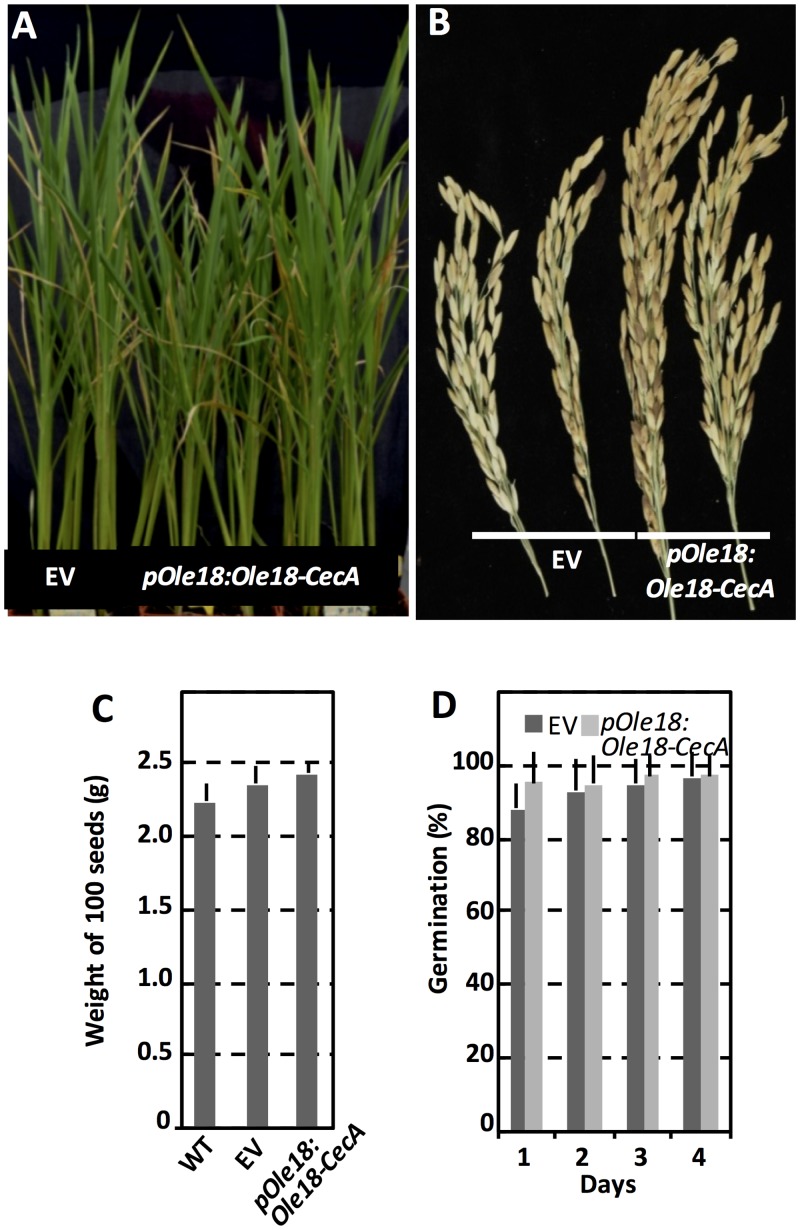
Phenotype of transgenic rice lines accumulating the Ole18-CecA fusion protein. (A) Phenotypic appearance of transgenic rice plants 60 days after sowing. (B) Images of representative panicles from transgenic plants. (C) Average weight of 100 seeds per line. (D) Percentage of germinated seeds at 1 to 4 days after imbibition from empty vector (EV) and *pOle18*:*Ole18-CecA* transgenic plants. All results are representative of two independent assays, in which 2 WT, 3 EV and 5 *pOle18*:*Ole18-CecA* independent lines were analyzed. Bars correspond to SD.

We also investigated whether the expression of *Ole18-CecA* driven by its own promoter has an effect on the growth and development of the rice plants. The *pOle18*:*Ole18-CecA* rice plants showed a normal phenotypic appearance during the vegetative phase (Fig 5A). They did not show a penalty in grain yield (Fig 5B), and seeds showed a similar weight to wild-type seeds or the empty vector seeds (Fig 5C). Moreover seeds accumulating the Ole18-CecA fusion protein germinated at the same rate and timing as empty vector seeds (Fig 5D), indicating that the presence of fusion protein in OBs has not a negative impact on seed viability and seedling growth. All together, these results demonstrate that the expression of the *pOle18*::*Ole18-CecA* did not alter the fitness of the rice plant.

## Discussion

The present study shows that the antimicrobial peptide CecA can be efficiently produced in rice seeds using the oleosin protein as a carrier, and that it can be easily recovered by a simple purification procedure. The fusion of CecA to the rice Ole18 protein resulted in a recombinant protein that properly targeted OBs, but did not exhibit biological activity at least while immobilized onto the OBs. Thus, the fusion of CecA to the oleosin conferred stability to the antimicrobial peptide by sequestering it within the OB compartment, and reducing its potential toxicity to the host cells. As a result, high accumulation levels of CecA were reached in plant tissues, measured on average at 38 μg/g seed.

High yields of recombinant proteins have been reported in rice seeds. For instance, values as high as 9.2 mg per gram of rice flour were reached for the human lyzozyme protein proving the synthesis capacity of rice as a host system [30]. However, for small peptides lower yields were obtained, either when produced as fusion to carrier proteins, or as tandem repeats, such as the Cry j I and Cry j II allergen peptides (as seven tandem repeats or fused to the soybean seed storage protein glycinin or to the cholera toxin B) [24,53,54], the tolerogen 3 Crp (fused to GluA) [55], the type II-collagen tolerogenic peptide (tandem of four monomers fused to GluA) [56], the TCP7 tolerogen [27], the allergen Derp1 [57], or the novokinin (as tandem repeats or fused to GluA or GluC) [58]. The levels of CecA produced with the oleosin fusion strategy (8 nmols/g of seed) are in the average of the above mentioned reports (0.03 to 10 nmols/g of seed), and are higher than when produce in the rice seed protein bodies as an independent peptide (1.25 nmols/g of seed). Note that these values are given in nmols/g instead of μg/g which takes into account the size of the peptide in relation to the fusion protein.

These increased accumulation levels are even more relevant since the endosperm occupies most of the volume of the rice grain (90%), whereas the OBs are restricted to the embryo and aleurone layers occupying only a very small portion of the rice seed (10%). That is, we are reporting much higher accumulation levels on a much smaller portion of the seed volume. It would be interesting to explore the efficiency of CecA production as an oleosin fusion protein in oil crops such as safflower, sesame, rapeseed, soybean or sunflower, previously used to produce pharmaceutical oleosin fusion proteins [43–45], because these plant species have higher OB content than the starchy rice seeds, and consequently higher accumulation levels can be expected. Our proof-of-concept study in rice seeds, nonetheless, allows the comparison among different production strategies for CecA: the production as an independent peptide in PBs [35] and the production as a fusion protein in OBs. Our results indicate that in the case of small bioactive molecules, such as the CecA, strategies based on their subcellular compartmentalization and their inactivation by fusing to carrier proteins improve their accumulation levels.

In this study, the expression of the recombinant *Ole18-CecA* gene was driven by its own *Ole18* promoter leading to fusion protein accumulation in the embryo and the aleurone layers of the rice seed. This distribution pattern matches the one described for the *GUS* reporter gene expressed under the control of the *Ole18* promoter, the GUS activity being restricted to the embryo and the aleurone layers, and excluded from seed endosperm and vegetative tissues [59]. Our results demonstrate the usefulness of the rice *Ole18* promoter to drive strong and tissue-specific expression of the *Ole18-CecA* recombinant gene. Limiting the accumulation of recombinant proteins to seeds has the advantage of not interfering with the vegetative growth of the plant [22]. In this sense, transgenic rice plants accumulating the recombinant Ole18-CecA protein in seed OBs showed an apparently normal vegetative growth. Moreover, the presence of this amphiphatic and strongly cationic CecA peptide on the surface of the OBs seems not to interfere with their functions during pollen and seed development, the two plant tissues where oleosins accumulate [60]. Indeed, the seed setting rate was not reduced in the transgenic plants, as well as seed filling, since they reached the same weight and morphology than wild-type seeds. Moreover, the seeds mobilizing the storage lipids of the CecA-containing OBs during germination had similar vigor and viability than wild-type seeds. These results indicate that limiting the production of the antimicrobial peptide to the seed OBs avoids negative effects on the host plant fitness.

The accumulation of the recombinant protein in OBs has the additional advantage of facilitating the purification and the recovery of bioactive CecA. OBs and their associated proteins including the CecA fusion protein were easily isolated from other seed components by flotation centrifugation. The CecA peptide was then released precisely and efficiently by proteolytic cleavage of the linker sequence between the oleosin18 and the CecA in the fusion protein immobilized on the intact OBs under non-denaturizing conditions. Finally, CecA was recovered in the soluble fraction by simple separation by flotation centrifugation of the OBs. The recovered fraction showed lytic activity against bacterial cells, proving the biological functionality of the *in planta* produced CecA. The efficiency of this production system was also demonstrated by the potency of the recovered products as antimicrobials at very low micromolar concentrations against the target phytopathogen *D*. *dadantii*. This is a simple purification procedure that results in a substantial enrichment of the AMP from the rest of seed proteins. This degree of purification may be good enough depending on the intended use of the peptide, for instance for crop protection applications. However, applications in medicine might require purification to homogeneity and removal of the added TEV protease. This difficulty might be solved by adding the TEV immobilized also in the OBs which could simplify and reduce the purification costs of the AMPs from the plant material. Evidences support that endoproteases may be active when immobilized in OBs [42,61–64]. Thus, CecA might be purified to homogeneity by simple liquid-liquid phase centrifugation without the need of chromatography, clearly reducing the downstream purification costs. Therefore, targeting the OB represents an economical strategy for the production and purification of this antimicrobial peptide.

High manufacturing cost is one of the major obstacles to the wide application of AMPs. The typical cost of peptides ranges between $100 and $600 per gram, which is much higher than that of conventional antibiotics [9]. Chemical synthesis, although very efficient, is a complex and costly process [65]. The biotechnological production can be less expensive, but their antimicrobial nature poses difficulties to the use of microbial-based systems. The fusion of AMPs to different carrier proteins has been reported as an effective strategy to mask the lethal effect of these peptides towards the host [66]. However, the current cost remains far from commercially acceptable. The production system explored in this study is based on rice seeds, having the advantages of low production costs, high yield capacity, easy scalability, lower risk of contamination with human pathogens, long-term stability of the recombinant peptide during storage at room temperature, thereby eliminating the need for cold-chain transportation and storage and decoupling of production and processing cycles [23,45]. In addition, the use of a rice oleosin as a new carrier protein of AMPs is shown to facilitate the recovery of these strongly cationic and amphipathic peptides that tend to interact with many cellular components. The CecA targeting to OBs through the fusion to the oleosin carrier protein results in specific accumulation in the embryos and aleurone layers of the rice grains. These tissues are easily separated during rice milling to obtain the white refined grain, and remain in the by-product or rice bran [67]. Thus, downstream purification would be favored using the recombinant protein-enriched rice bran, easily obtained as the starting plant material. All together, this proof-of-concept study demonstrates that rice OB targeting is a useful strategy for efficient production of CecA, and presumably the production system here described can be potentially extended to other valuable bioactive peptides. The use of rice bran for the production of AMPs could add an extra value to this by-product by assisting their exploitation in crop protection, food preservation and medical applications.

## Supporting Information

S1 Fig Alignment between the *18 kDa Oleosin* promoter sequence isolated from indica IR36 (Ole18) and japonica Nipponbare (AY427563) rice varieties. Identical nucleotides are indicated by stars, nucleotide changes by red color and deletion-insertion by hyphens. The 5´untranslated region is indicated in blue color. Restriction sites used for cloning (*EcoR*I and *Bsm*I) are highlighted in yellow, and the sequence of primers used for amplification are underlined.
(PDF)

S2 Fig Confirmation of transgene insertion in the genome of transgenic rice plants. (A) Diagram of the transgene inserted in transgenic lines. Arrows indicate the position of the specific oligonucleotides used for PCR amplification. (B-C) PCR analysis of genomic DNA purified from leaves of wild-type (WT) or transgenic lines in Ariete (B) or Senia (C) cultivars carrying the empty vector (EV) or the indicated transgene. Plasmidic DNA was used as a positive control (+), no DNA sample as a negative control (-). The size of amplified fragments showed full length transgene insertion.
(PDF)

S3 Fig Ole18-CecA fusion protein accumulates in seed OBs of *pOle18*:*Ole18-CecA* Senia rice transgenic lines. OB protein extracts were prepared from mature rice seeds of wild-type (WT), empty vector (EV) or the indicated *pOle18*:*Ole18-CecA* lines (T3 homozygous lines) and subjected to SDS-PAGE (35 μg per lane). Western blot analysis was performed using the anti-cecropin A (upper panel) or anti-Oleosin18 (middle panel) antibodies. Synthetic cecropin A peptide (0.16 μg) was used as a positive control. Lower panel shows Stain free gel of protein samples. Molecular weight markers are indicated on the left in kDa.
(PDF)

S1 Table Primers used for cloning and for transgene detection in this study. (PDF)

## Abbreviations

AMP: antimicrobial peptide
CecA: Cecropin A
MRM: multiple reaction monitoring
MS: mass spectrometry
Nos: Nopaline synthase
OB: oil body
Ole: oleosin
SD: standard desviation
SEM: standard error of the mean
TEV: Tobacco Etch Virus
