# Supplementary material for: Production of Biologically Active Cecropin A Peptide in Rice Seed Oil Bodies
Source: PLoS One. 2016 Jan 13;11(1):e0146919. doi: 10.1371/journal.pone.0146919 (PMC4711921; doi:10.1371/journal.pone.0146919)
Supplement: S1 Fig — Identical nucleotides are indicated by stars, nucleotide changes by red color and deletion-insertion by hyphens. The 5´untranslated region is indicated in blue color. Restriction sites used for cloning (EcoRI and BsmI) are highlighted in yellow, and the sequence of primers used for amplification are underlined. (PDF) [file pone.0146919.s001.pdf]

```
Ole18          GAATTCGATGGTCAGCCAATACATTGATCCGTTGCCAATCATGCAAAGTATTTTGGCTGT 60
AY427563       ------GATGGTCAGCCAATACATTGATCCGTTGCCAATCATGCAAAGTATTTTGGCTGT 54
                     ******************************************************

Ole18          GGCCGAGTGCCGGAATTGATAATTGTGTTCTGACTAAATTAAATGACCAGAAGTCGCTAT 120
AY427563       GGCCGAGTGCCGGAATTGATAATTGTGTTCTGACTAAATTAAATGACCAGAAGTCGCTAT 114
               ************************************************************

Ole18          CTTCCAATGTATCCGAAACCTGGATTAAGCAATCCTGTTCTGTTCTCTAGCCCCTCCTGC 180
AY427563       CTTCCAATGTATCCGAAACCTGGATTAAACAATCCTGTTCTGTTCTCTAGCCCCTCCTGC 174
               **************************** *******************************

Ole18          ATGGCCGGATTGTTTTTTTGACATGTTTTCTTGACTGAGGCCTGTTTGTTCTAAACTTTT 240
AY427563       ATGGCCGGATTGTTTTTTTGACATGTTTTCTTGACTGAGGCCTGTTTGTTCTAAACTTTT 234
               ************************************************************

Ole18          TCTTCAAACTTTTAACTTTTTCATCACATCAGAACTTTTCTACACACATAAACTTTTAAC 300
AY427563       TCTTCAAACTTTTAACTTTTTCATCACATCAGAACTTTTCTACACATATAAACTTTTAAC 294
               ********************************************** *************

Ole18          TTTTCTGTCACATCGTTCCAATTTCAATCAAACTTTTAATTTTGGCTTGAACTAAACACA 360
AY427563       TTTTCCGTCACATCGTTCCAATTTCAATCAAACTTTCAATTTTGGCGTGAACTAAACACA 354
               ***** ****************************** ********* *************

Ole18          CCCTGAGTCTTTTATTGCTCCTCCATACGGGTTGGCTGGTTGAGAATAGGTATTTTCAGA 420
AY427563       CCCTGAGTCTTTTATTGCTCCTCCGTACGGGTTGGCTGGTTGAGAATAGGTATTTTCAGA 414
               ************************ ***********************************

Ole18          GAGAAAATCTGGATATTGGGAGGAGGAACTTGGCATGAATGGCCACTATATTTAGAGCAA 480
AY427563       GAGAAAATCTAGATATTGGGAGGA---ACTTGGCATGAATGGCCACTATATTTAGAGCAA 471
               ********** *************   *********************************

Ole18          TTCTACGGTCTTTGAGGAGGTACCATGAGGTACCAAAATTTTAGTGTAAATTTTAGTATC 540
AY427563       TTCTACGGTCCTTGAGGAGGTACCATGAGGTACCAAAATTTTAGTGTAAATTTTAGTATC 531
               ********** *************************************************

Ole18          T----------------------------------------------------------- 541
AY427563       TCATTATAACTAGGTATTATGAGGTACCAAATTTACAATAGAAAAAATAGTACTTCATGG 591
               *

Ole18          -----TCTTAAGGACCGTAAAATTGCTCCTATATTTAAGGG-ATGTTTATATCTATCCAT 595
AY427563       TACTTTCTTAAGTACCGTAAAATTGCTCCTATATTTAAGGGGATGTTTATATCTATCCAT 651
                    ******* **************************** ******************

Ole18          ATCCATAATTTGATTTTGATAAGAAAAAATGTGAGCACACCAAGCATGTCCATGACCTTG 655
AY427563       ATCCATAATTTGATTTTGATAAGAAAAAATGTGAGCACACCAAGCATGTCCATGACCTTG 711
               ************************************************************

Ole18          CACTCTTGGCTCACTCGTCAACTGTGAAGAACCTAAAAAATGCTCAATATAGCTACAGGT 715
AY427563       CACTCTTGGCTCACTCGTCAACTGTGAAGAACCTCAAAAATGCTCAATATAGCTACAGGT 771
               ********************************** *************************

Ole18          GCCTGAAAAAATAACTTTAAAGTTTTGAACATCGATTTCACTAAACAACAATTATTATCT 775
AY427563       GCCTGAAAAAATAACTTTAAAGTTTTGAACATCGATTTCACTAAACAACAATTATTATCT 831
               ************************************************************

Ole18          CCCTCTGAAATGTTGCTACCTAAGATGATAGTTTAGAACTCTAGAATCATTGTCGGCGGA 835
AY427563       CCCTCTGAAA-------------GATGATAGTTTAGAACTCTAGAATCATTGTCGGCGGA 878
               **********             *************************************

Ole18          GAAAGTAAATTATTTTCCCCAAATTTCCAGCTATGAAAAAACCCTCACCAAACACCATCA 895
AY427563       GAAAGTAAATTATTTTCCCCAAATTTCCAGCTATGAAAAAACCCTCACCAAACACCATCA 938
               ************************************************************

Ole18          AACAAGAGTTCACCAAACCGCCCATGCGGCCATGCTGTCACGCAACGCACCGCATTGCCT 955
AY427563       AACAAGAGTTCACCAAACCGCCCATGCGGCCATGCTGTCACGCAACGCACCGCATTGCCT 998
               ************************************************************

Ole18          GATGGCCGCTCGATGCATGCATGCTTCCCCGTGCACATATCCGACAGACGCGCCGTGTCA 1015
AY427563       GATGGCCGCTCGATGCATGCATGCTTCCCCGTGCACATATCCGACAGACGCGCCGTGTCA 1058
               ************************************************************

Ole18          GCGAGCTCCTCGACCGACCTGTGTAGCCCATGCAAGCATCCACCCCCGCCACGTACACCC 1075
AY427563       GCGAGCTCCTCGACCGACCTGTGTAGCCCATGCAAGCATCCACCCCCGCCACGTACACCC 1118
               ************************************************************

Ole18          CCTCCTCCTCCCTACGTGTCACCGCTCTCTCCACCTATATATGCCCACCTGGCCCCTCTC 1135
AY427563       CCTCCTCCTCCCTACGTGTCACCGCTCTCTCCACCTATATATGCCCACCTGGCCCCTCTC 1178
               ************************************************************

Ole18          CTCCCATCTCCACTTCACCCGATCGCTTCTTCTTCTTCTTCGTTGCATTCATCTTGCTAG 1195
AY427563       CTCCCATCTCCACTTCACCCGATCGCTTCTTCTTCTTCTTCGTTGCATTCATCTTGCTAG 1238
               ************************************************************

Ole18          CTCGCTTAGCA 1206
AY427563       CTAGCTTAGCA 1249
               ** ********
```
