# Supplementary figures and images for: Production of Biologically Active Cecropin A Peptide in Rice Seed Oil Bodies
Source: PLoS One. 2016 Jan 13;11(1):e0146919. doi: 10.1371/journal.pone.0146919 (PMC4711921; doi:10.1371/journal.pone.0146919)

**A**

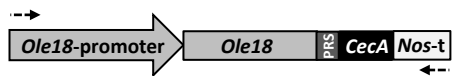

**B**

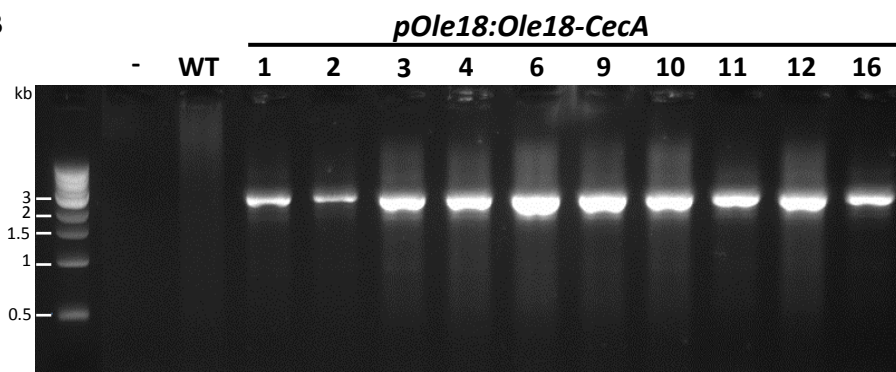

**C**

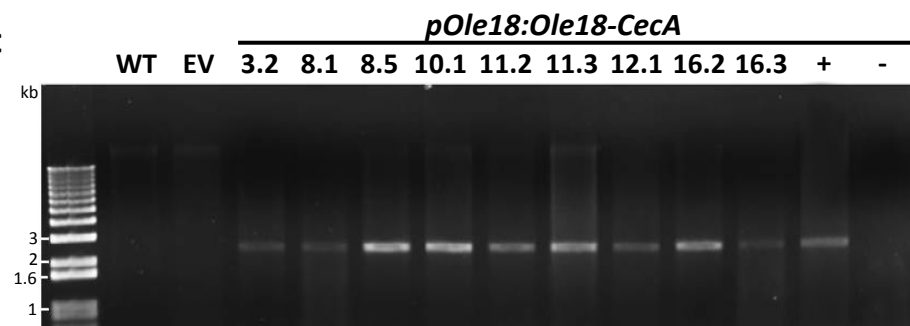

Supplement: S2 Fig — (A) Diagram of the transgene inserted in transgenic lines. Arrows indicate the position of the specific oligonucleotides used for PCR amplification. (B-C) PCR analysis of genomic DNA purified from leaves of wild-type (WT) or transgenic lines in Ariete (B) or Senia (C) cultivars carrying the empty vector (EV) or the indicated transgene. Plasmidic DNA was used as a positive control (+), no DNA sample as a negative control (-). The size of amplified fragments showed full length transgene insertion. (PDF) [file pone.0146919.s002.pdf]

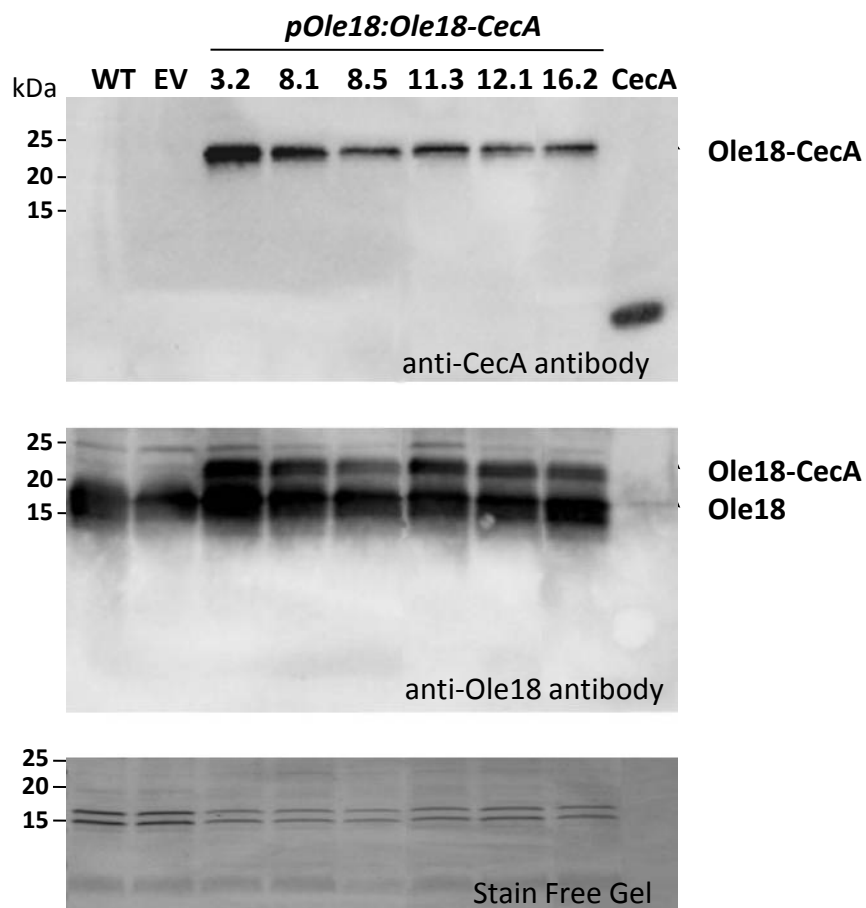

Supplement: S3 Fig — OB protein extracts were prepared from mature rice seeds of wild-type (WT), empty vector (EV) or the indicated pOle18:Ole18-CecA lines (T3 homozygous lines) and subjected to SDS-PAGE (35 μg per lane). Western blot analysis was performed using the anti-cecropin A (upper panel) or anti-Oleosin18 (middle panel) antibodies. Synthetic cecropin A peptide (0.16 μg) was used as a positive control. Lower panel shows Stain free gel of protein samples. Molecular weight markers are indicated on the left in kDa. (PDF) [file pone.0146919.s003.pdf]
