# Supplementary material for: Production of Biologically Active Cecropin A Peptide in Rice Seed Oil Bodies
Source: PLoS One. 2016 Jan 13;11(1):e0146919. doi: 10.1371/journal.pone.0146919 (PMC4711921; doi:10.1371/journal.pone.0146919)
Supplement: S1 Table — (PDF) [file pone.0146919.s004.pdf]

Table S1. Primers used for cloning and for transgene detection in this study.

Name	Sequence	Restriction site
Ole18prom_fwd	5´GGGAATTCGATGGTCAGCCAATACATTGATCCGTT3´	*EcoR* I
Ole18prom_rev	5´TGCTAAGCTAGCTAGCTAGCAAGATGAATGCAACGAAGA3´	*Bsm* I
Ole18cds1_fwd	5´GGCGTTGCATTCATCTTGCTAGCTAGCTTA3´	*Bsm* I
Ole18cds2_rev	5´CCCGAGGATGTCTTGGTG3´	
Ole18cds3_rev	5´CCTAGAGGTTCTCGGTGGTGGGCGAGGATGTCTTGGTG3´	
Ole18cds4_rev	5´CCAAGCTTCCACTTGCTCTGGAAGTAGAGGTTCTCGGTGGTGGG3´	*Hind* III
Nosterm_rev	5´CCGATCTCGTTTGACAGCTTATCATCGGATCTA	*Sac* I
